# Prenatal androgen treatment impairs the suprachiasmatic nucleus arginine-vasopressin to kisspeptin neuron circuit in female mice

**DOI:** 10.3389/fendo.2022.951344

**Published:** 2022-08-05

**Authors:** Bradley B. Jamieson, Aleisha M. Moore, Dayanara B. Lohr, Simone X. Thomas, Lique M. Coolen, Michael N. Lehman, Rebecca E. Campbell, Richard Piet

**Affiliations:** ^1^ Centre for Neuroendocrinology and Department of Physiology, University of Otago, Dunedin, New Zealand; ^2^ Brain Health Research Institute and Department of Biological Sciences, Kent State University, Kent, OH, United States

**Keywords:** GnRH, LH surge, circadian, androgen receptor, PCOS, tract-tracing, electrophysiology

## Abstract

Polycystic ovary syndrome (PCOS) is associated with elevated androgen and luteinizing hormone (LH) secretion and with oligo/anovulation. Evidence indicates that elevated androgens impair sex steroid hormone feedback regulation of pulsatile LH secretion. Hyperandrogenemia in PCOS may also disrupt the preovulatory LH surge. The mechanisms through which this might occur, however, are not fully understood. Kisspeptin (KISS1) neurons of the rostral periventricular area of the third ventricle (RP3V) convey hormonal cues to gonadotropin-releasing hormone (GnRH) neurons. In rodents, the preovulatory surge is triggered by these hormonal cues and coincident timing signals from the central circadian clock in the suprachiasmatic nucleus (SCN). Timing signals are relayed to GnRH neurons, in part, *via* projections from SCN arginine-vasopressin (AVP) neurons to RP3V^KISS1^ neurons. Because rodent SCN cells express androgen receptors (AR), we hypothesized that these circuits are impaired by elevated androgens in a mouse model of PCOS. In prenatally androgen-treated (PNA) female mice, SCN *Ar* expression was significantly increased compared to that found in prenatally vehicle-treated mice. A similar trend was seen in the number of *Avp*-positive SCN cells expressing *Ar*. In the RP3V, the number of kisspeptin neurons was preserved. Anterograde tract-tracing, however, revealed reduced SCN^AVP^ neuron projections to the RP3V and a significantly lower proportion of RP3V^KISS1^ neurons with close appositions from SCN^AVP^ fibers. Functional assessments showed, on the other hand, that RP3V^KISS1^ neuron responses to AVP were maintained in PNA mice. These findings indicate that PNA changes some of the neural circuits that regulate the preovulatory surge. These impairments might contribute to ovulatory dysfunction in PNA mice modeling PCOS.

## 1 Introduction

Polycystic ovary syndrome (PCOS) is a complex and prevalent endocrine disorder, affecting up to 20% of women of reproductive age, and is the most common cause of anovulatory infertility (1, 2). PCOS is associated with clinical and/or biochemical signs of elevated androgen secretion, polycystic ovaries and oligo- or anovulation. The presence of two out of three of these symptoms is the currently accepted criterion for diagnosing PCOS (3, 4). Although the reproductive symptoms of PCOS are expressed in the periphery, some of these dysfunctions may have a central origin. Indeed, ovarian dysfunction in PCOS is thought to be brought about by impaired feedback regulation of the pulsatile gonadotropin-releasing hormone (GnRH) neuron drive on anterior pituitary gonadotropin release, causing follicular arrest and elevated androgen secretion (1, 5).

The etiology of PCOS is not well understood but likely results from the combination of genetic, developmental and environmental factors (6). It has been suggested that prenatal and/or early postnatal exposure to excess androgens might contribute to the abnormal programming of the hypothalamic-pituitary gonadal (HPG) axis and result in endocrine perturbations and ovulatory dysfunction in adult PCOS patients (7). This is supported by preclinical studies in which exposure to high androgens *in utero* induces PCOS-like symptoms in adult females of many species (8). Consistent with the idea that PCOS is associated with central dysregulation of the HPG axis, some of the reproductive and endocrine phenotypes induced by exposure to excess androgens are prevented by neuron-specific androgen receptor (AR) knockout in mice (9). In addition, we and others have reported that prenatal androgen exposure (PNA) is associated with anatomical, functional and molecular changes within the GnRH neuronal network, including to the kisspeptin (KISS1) neurons, in adulthood (10–19). Although arcuate nucleus kisspeptin neurons, critical in generating pulsatile luteinizing hormone (LH) secretion (20–23), are likely impaired by endocrine dysregulations associated with PCOS, the kisspeptin neurons involved in the preovulatory gonadotropin surge mechanism may also be affected, contributing to impaired ovulatory function in PCOS. How this might occur, however, has not been fully investigated.

In many species, including primates, estrogen positive feedback signals driving the preovulatory GnRH/LH surge are detected and relayed to the GnRH neurons, at least partially, by kisspeptin neurons located in the preoptic area (POA) of the hypothalamus (24–35). In female rodents, the LH surge occurs just before the daily onset of activity, ensuring optimal coordination of ovulation and sexual behavior (36–40). Generation and timing of the surge are dependent on axonal outputs from the suprachiasmatic nucleus (SCN) of the hypothalamus (41–43), where the central circadian clock is located (44–46). GnRH neurons receive direct innervation from vasoactive intestinal peptide (VIP)-expressing SCN neurons (47), which may drive their activity (48–50). Upstream of GnRH neurons, kisspeptin neurons in the rostral periventricular area of the third ventricle (RP3V^KISS1^) are innervated by arginine vasopressin (AVP)-expressing SCN neurons (51–53), which drive their activity on proestrus *via* the release of AVP (53). Although RP3V^KISS1^ neurons express the gene that encodes AR (*Ar*) (54), prior studies have reported little effect of PNA on *Kiss1* expression in the RP3V or on RP3V^KISS1^ neuron numbers (18, 19, 55). On the other hand, AR is found in the rodent SCN (56) and androgen treatment of ovariectomized female rats upregulates its expression to levels comparable to those found in the male SCN (56, 57). It is, therefore, conceivable that PNA might affect androgen signaling in the SCN, thereby altering the circuits that generate the preovulatory surge.

We hypothesized here that PNA alters the SCN to RP3V^KISS1^ neuron circuit in adult female mice. Using RNAscope^®^, we observed that *Ar* expression is upregulated in the SCN of adult PNA female mice. Adeno-associated virus (AAV)-mediated conditional anterograde tract-tracing revealed that this is associated with fewer projections from SCN^AVP^ neurons to RP3V^KISS1^ neurons. However, we observed that the function of the SCN^AVP^→RP3V^KISS1^ neuron circuit was not measurably changed on diestrus. This suggests that PNA might prevent the estrous cycle-dependent plasticity in the output of this circuit, which drives kisspeptin neuron firing on proestrus (53). We propose that these changes negatively affect the circuits that initiate the preovulatory surge and, therefore, contribute to ovulatory dysfunction in female PNA mice.

## 2 Materials and methods

### 2.1 Animals and prenatal androgen treatment

All animals used in this study were adult female mice (3 – 6 months old), including C57BL/6J mice (Jackson Laboratory stock #000664), mice that express the Cre recombinase enzyme (cre) in AVP neurons (Avp-cre; Jackson Laboratory stock #023530), mice that express humanized *renilla* green fluorescent protein (hrGFP) in kisspeptin neurons (Kiss1-hrGFP; Jackson Laboratory stock #023425) (58) and Avp-cre mice that also express Kiss1-hrGFP (Avp-cre:Kiss1-hrGFP). Avp-cre mice were generated by mating Avp-cre^+/-^ with Avp-cre^-/-^ parents. Avp-cre:Kiss1-hrGFP mice were obtained from matings of Avp-cre^+/-^ with Kiss1-hrGFP positive (Kiss-hrGFP^pos^) parents or from matings of Avp-cre^+/-^:Kiss1-hrGFP^pos^ with Kiss1-hrGFP^pos^ parents. Kiss1-hrGFP mice were offspring of these matings that were Avp-cre^-/-^ and Kiss1-hrGFP^pos^.

To induce PCOS-like symptoms in female mice, we used the well-established and well-characterized PNA model (14, 59). Time-mated pregnant dams were injected subcutaneously with either 100 µL of sesame oil containing dihydrotestosterone (DHT, 250 µg) to generate PNA offspring or 100 µL of sesame oil alone (as a vehicle control, VEH) on gestational days 16 to 18. The female offspring were used in experiments. Mice were group-housed with littermates under controlled temperature (22 ± 2°C) and lighting (12 h light/dark cycle) conditions with *ad libitum* access to food and water. Mice were assigned to experiments based on their genotype and treatment. All experiments were approved by the Kent State Institutional Animal Care and Use Committee and by the University of Otago Animal Ethics Committee.

### 2.2 Determination of estrous cycle stage

Estrous cycle stage was determined by vaginal lavage (4 μL H_2_O) or by collecting vaginal cells using a small metal loop. This was carried out between zeitgeber time (ZT) 2-4. Vaginal smears were stained with toluidine blue or methylene blue. Cytology was examined under light microscopy at 10× magnification to identify estrous cycle stage (60). As reported previously (14, 59), PNA mice did not have regular cycles. In a subset of four PNA and four VEH mice monitored over a 14 day period, VEH mice exhibited clear estrous cycles, spending on average 25.0 ± 2.1% of the cycle in diestrus and 25.0 ± 4.6% in proestrus, whereas PNA mice spent 76.8 ± 8.4% of their estrous cycles in diestrus. We never detected a proestrous stage in PNA mice.

### 2.3 Stereotaxic surgery and viral vector injection

Avp-cre and Avp-cre:Kiss1-hrGFP mice were anesthetized with isoflurane and placed in a stereotaxic apparatus (Kopf Instruments, Tujunga, CA, USA). Viral vectors were bilaterally injected (400 nL/side) at a rate of 100 nL/min into the SCN (from bregma: 0.5 mm anterior, ± 0.2 mm lateral, 5.8 mm in depth from surface of the skull) with a 1 mL Hamilton syringe. The needle was left *in situ* for 3 min before, and 10 min after injections. Viral vectors were AAV-DJ-EF1-DIO-mCherry (AAV-DIO-mCherry; 5.05×10^13^ GC/mL) and AAV-DJ-EF1-DIO-hChR2^(E123T/T159C)^-p2a-mCherry-WPRE (AAV-DIO-ChR2-mCherry; 1.60×10^13^ GC/mL) (61, 62).

### 2.4 Multiplex fluorescent *in situ* hybridization

RNAscope^®^ (ACDBio, Newark, CA, USA) multiplex fluorescent ISH was used to assess the expression of *Ar* in the SCN. C57BL/6J female VEH (n=5) and PNA (n=5) mice were killed by intraperitoneal (ip) pentobarbital overdose (ZT6-8) and perfused through the heart with 4% paraformaldehyde (PFA). Brains were extracted and incubated at 4°C for 24 hours in the same fixative. Brains were then sunk in 10%, 20% and 30% sucrose in 0.1M phosphate buffer saline (PBS), rapidly frozen in OCT and stored at -80°C until cryosectioning coronal sections onto superfrost charged slides at 12 μm thickness. Sections were stored at -80°C until use. The assay was performed following the manufacturer’s instructions (ACDBio; 323100-USM) with RNAscope ® probes designed to detect *Ar* (Cat. #316991) and *Avp* (Cat. #401391), which were labelled with red (Cy3) and green (Fluorescein) TSA Plus fluorophores, respectively, after signal amplification steps. Finally, cell nuclei were counterstained using 4′,6-diamidino-2-phenylindole (DAPI; ACDBio) and slides were coverslipped using ProLong Gold Antifade Mountant (Fisher Scientific, Cat. #P36930).

### 2.5 Immunohistochemistry

Avp-cre mice injected with AAV-DIO-mCherry were killed by pentobarbital overdose (ip; ZT4-6) and perfused through the heart with 4% PFA. 40 µm thick coronal brain sections were cut through the RP3V and SCN, of which one in three slices were processed for free-floating IHC. Antibodies used were rabbit anti-mCherry (1:5000; AbCam, Cat. #ab167453) visualized with donkey anti-rabbit AlexaFluor-568 (1:200; ThermoFisher Scientific, Cat. #A10042), and sheep anti-kisspeptin (1:1000; Gift from Prof. Alain Caraty, AC053) visualized with biotinylated donkey anti-sheep (1:1000; ThermoFisher Scientific, Cat. #A16045) revealed by streptavidin AlexaFluor-488 (1:1000; ThermoFisher Scientific, Cat. #S32354).

### 2.6 Confocal microscopy and image analysis

For ISH, two sections containing the SCN were imaged per animal using an Olympus FV3000 confocal microscope. A 20x objective was used to enable imaging of the unilateral SCN. Optical sections with a 2 μm step size were acquired using 405 nm, 488 nm and 550 nm lasers. For IHC, brain slices were imaged using an inverted Nikon A1R confocal microscope (Nikon Instruments Inc., Tokyo, Japan), with lasers of 488 and 543 nm wavelengths. Z-stack images were taken through the RP3V and SCN. Images were taken at 10× (1 µm Z-step, 1 AU pinhole, 0.5 NA) or 40× (0.5 µm Z-step, 1 AU pinhole, 0.9 NA) magnification. Images were analyzed offline using the FIJI software (63).

#### 2.6.1 SCN cell *Ar* gene expression analysis

*Ar* and *AVP* mRNA expression was quantified in DAPI-labelled cells in the unilateral SCN of two sections per animal. Cells within the SCN were deemed positive for *Ar* or *Avp* expression when three or more mRNA puncta overlayed DAPI. The number of *Ar*-only cells, *Avp*-only cells and cells coexpressing *Avp* and *Ar* were counted, and the percentages of SCN *Avp*-positive neurons co-expressing *Ar* and of *Ar*-positive cells co-expressing *Avp* were calculated. In addition, the number of *Ar* puncta within AVP and non-AVP cells was quantified. For all analyses, calculations were averaged for each female and the mean coexpression levels calculated per group.

#### 2.6.2 Axonal projection analyses

mCherry-immunoreactive (ir) projection fiber density was measured on two sets of 10× Z-stack images through the RP3V, including the anteroventral periventricular nucleus (AVPV) and the periventricular preoptic nucleus (PVpo). Maximum projection images were binarized (threshold: 1-1.5%). A region of interest (ROI) extending from the ventral side of the section to the dorsal edge of the third ventricle and 100 μm lateral to the wall of the third ventricle was drawn to include the RP3V in each maximum projection image. The percentage of area covered by mCherry-ir fluorescent pixels (pixel density) was measured in each ROI. This value was averaged across the two RP3V sections in each mouse. To estimate the extent of transfection in the SCN, pixel density was measured within two ROIs delineating the bilateral SCN in a single slice through the middle of its rostro-caudal extent. Measurements were carried out on both sides of the brain. As previously (53), injections that resulted in mCherry-ir covering greater than 5% of the area of at least one ROI were considered successful. For quantifying innervation of the RP3V of VEH and PNA mice, only those animals with successful uni- or bilateral transfection were included, and innervation reported on the side of the brain ipsilateral to the most transfected SCN. In analyses correlating RP3V innervation to SCN transfection in VEH or PNA mice, values obtained on both sides of the brain of all mice in each group were included (n = 18 hemibrains in 9 mice each).

#### 2.6.3 Cell counting and close apposition quantifications

RP3V kisspeptin-ir neurons were imaged at 40× magnification in the same sections as above. Kisspeptin-ir somata were counted bilaterally in two sets of Z-stacks containing the AVPV or PVpo. Close appositions between mCherry-ir fibers and kisspeptin-ir somata were quantified on the side ipsilateral to the most transfected SCN. Individual kisspeptin-ir somata were digitally magnified 4 times and inspected in successive single confocal planes of the Z-stack to determine the presence of appositions between a red fiber and a green soma with no black pixel in-between, as described previously (16, 53, 64). The proportion of kisspeptin-ir neurons with at least one apposition, expressed as a percentage, was calculated for the two RP3V slices and averaged in each mouse.

### 2.7 Brain slice preparation and electrophysiology

Avp-cre:Kiss1-hrGFP and Kiss1-hrGFP mice were killed by cervical dislocation (ZT3-5), decapitated and their brains quickly removed as described previously (53). Coronal brain slices (200 µm thick) containing the RP3V or the SCN were cut using a vibratome (VT1000S, Leica) in an ice-cold slicing solution containing (in mM): 87 NaCl, 2.5 KCl, 25 NaHCO_3_, 1.25 NaH_2_PO_4_, 0.5 CaCl_2_, 6 MgCl_2_, 25 glucose and 75 sucrose. Brain slices were left to incubate at 30°C for at least 1 hour in artificial cerebrospinal fluid (aCSF) containing (in mM): 120 NaCl, 3 KCl, 26 NaHCO_3_, 1 NaH_2_PO_4_, 2.5 CaCl_2_, 1.2 MgCl_2_ and 10 glucose. All solutions were equilibrated to pH 7.4 with a mixture of 95% O_2_/5% CO_2_. Individual slices were then placed under an upright epifluorescence microscope (Scientifica, UK) and constantly perfused (1.5 mL/min) with warm (~30°C) aCSF. In AAV-DIO-ChR2-mCherry injected mice, we first confirmed that the SCN was sufficiently transfected by visualizing mCherry expression using light-emitting diode (LED; CoolLED, UK) illumination (excitation 542-582 nm, emission 604-679 nm). RP3V GFP-expressing neurons were visualized using brief LED illumination (GFP: excitation 446-486 nm, emission 500-550 nm) and subsequently approached with recording electrodes using infrared differential interference contrast illumination. Because the ChR2 excitation spectrum overlaps that of GFP, low intensity LED illumination was used (~0.8 mW) and a waiting period of at least 10 min following identification of a GFP-expressing neuron was allowed before recordings started (32, 53). Recordings were made with borosilicate glass electrodes (tip resistance: 2-5 MΩ), pulled using a Model P-97 Flaming/Brown micropipette puller (Sutter Instruments, USA). Electrophysiological signals were recorded using a Multiclamp 700A or 700B amplifier (Molecular Devices, USA), filtered at 2 kHz, and digitized at 10 kHz using a Digidata 1322a or 1440a (Molecular Devices, USA). Signal acquisition and analysis was performed with pClamp 9 or 10 (Molecular Devices, USA). Action potential firing was recorded in voltage-clamp mode (no holding potential applied) in the loose-seal, cell-attached configuration (12-20 MΩ seal resistance). Glass microelectrodes were filled with aCSF and the recording configuration was achieved by applying the lowest amount of suction required to detect spikes. To activate ChR2, trains (20 Hz for 60 s) of LED blue light pulses (5 ms duration; 446-486 nm; ~14 mW) were driven by a Master-9 stimulus pattern generator (AMPI, Israel) and delivered to the slice through a 40× immersion objective (0.8 NA, Olympus). In experiments testing the effect of exogenous AVP on spontaneous firing, 500 nM AVP (Tocris-Bioscience, Cat. #2935) was bath applied for 2 minutes after a 2-minute baseline, before being washed out for at least 5 minutes. AVP was dissolved in water to a stock concentration, kept at -20°C until use, and diluted to the appropriate concentration in aCSF. All recordings were carried out between ZT4-10.

Loose seal, cell-attached recordings were analyzed in ClampFit (pClamp, Molecular Devices). Individual spikes, corresponding to action potentials, were detected using the threshold crossing method. Spike initiation time stamps were organized into 10 s bins and the mean firing rate calculated for each bin. In optogenetics experiments, baseline firing rate was the mean firing rate prior to stimulation, immediate response was the mean firing rate during light stimulation and delayed response was the mean firing rate in the bin with greatest deviation from baseline as well as during the two preceding and two following bins in the five minutes post-stimulation. For AVP bath applications, baseline firing rate was the mean firing rate prior to the application whereas the delayed effect was measured as above during the five minutes after the application. If mean firing rates changed by greater than twice the standard deviation of baseline firing (2×SD), this was recorded as an excitatory or inhibitory effect. For those recordings where mean firing rates did not change by greater than 2×SD, measurements were taken at the time when firing deviated the most from baseline during the relevant recording time periods (immediate or delayed).

### 2.8 Statistical analyses

Statistics were performed using Prism 9.0 (GraphPad). Data are presented in text and figures as mean ± SEM, or linear regression line of best fit ± 95% confidence interval. Comparisons between VEH and PNA were made using unpaired t-tests. Comparisons of linear regression slopes between VEH and PNA was carried out using ANCOVA. Within the VEH and PNA groups, comparisons between two time points were undertaken with paired t-tests while comparisons of more than two time points were carried out with one-way ANOVA. For electrophysiology experiments, proportions of responding cells were compared using chi-squared tests, with the number of individual recordings as the sample size. In RNAscope^®^ and tract-tracing experiments, proportions were compared with unpaired t-tests, and the number of animals was the sample size. Differences were considered statistically significant for a p-value less than 0.05.

## 3 Results

### 3.1 PNA increases *Ar* expression in SCN cells

**Figure 1:**
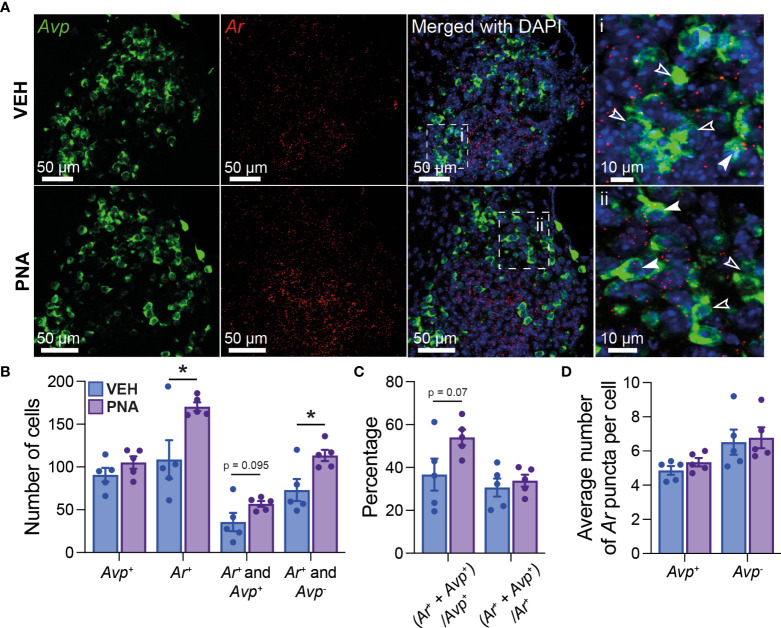
PNA increases *Ar* expression in the SCN of female mice. **(A)** (*Left*) Example confocal images of sections from VEH and PNA mice, showing expression of *Avp* (green) and *Ar* (red) mRNA in the SCN. (*Right*) DAPI counterstain merged with *Avp* and *Ar* staining revealed *Ar*-positive cells containing (filled arrowheads) or not (empty arrowheads) *Avp* mRNA. **(B)** Summary graph of the numbers of SCN cells expressing transcripts for *Avp* (*Avp* ^+^), *Ar* (*Ar* ^+^), *Ar* and *Avp (Ar* ^+^ and *Avp* ^+^ *)*, or *Ar* only (*Ar* ^+^ and *Avp* ^-^) in VEH and PNA mice. **(C)** Summary of the proportions of *Avp*-containing cells also expressing *Ar* [(*Ar* ^+^ + *Avp* ^+^)/*Avp* ^+^] and of *Ar*-containing cells also expressing *Avp* [(*Ar* ^+^ + *Avp* ^+^)/*Ar* ^+^] in the SCN of VEH and PNA mice. **(D)** Summary graph of the numbers of *Ar* particles in SCN cells with and without *Avp* expression. *p < 0.05 unpaired t-tests.

Using RNAscope^®^, we first quantified *Ar* mRNA expression in the SCN of five diestrous VEH and five diestrous PNA mice. *Ar* mRNA expression was seen throughout the SCN, although it appeared more concentrated in the core subdivision (**Figure 1A**). PNA significantly increased the total number of *Ar*-expressing SCN cells (VEH: 108.7 ± 22.54; PNA: 170.3 ± 5.06, p = 0.029 unpaired t-test; **Figures 1A, B**). The total number of *Avp*-expressing cells was similar in VEH (90.80 ± 8.08) and PNA mice (105.10 ± 7.24; p = 0.22 unpaired t-test). Interestingly, *Ar* particles were detected in subsets of *Avp*-expressing SCN neurons in both groups of mice (**Figure 1A**). There tended to be a greater number of *Avp*-expressing cells also containing *Ar* mRNA particles in PNA, but this did not reach significance (VEH: 35.7 ± 10.67; PNA: 56.9 ± 3.34; p = 0.095, unpaired t-test; **Figures 1A**, **B**). The number of cells expressing *Ar* but not *Avp*, however, was significantly greater in PNA (VEH: 73.0 ± 12.78; PNA: 113.4 ± 6.65; p = 0.023 unpaired t-test, **Figures 1A**, **B**). The proportion of *Avp*-expressing cells also expressing *Ar* tended to be higher in PNA (VEH: 38.68 ± 7.44%; PNA: 54.04 ± 3.66%; p = 0.07 unpaired t-test) but the proportion of *Ar* cells expressing *Avp* was unchanged (VEH: 30.64 ± 4.22%; PNA: 33.86 ± 2.76%; p = 0.54 unpaired t-test; **Figure 1C**). Lastly, numbers of *Ar* mRNA particles were not affected by PNA in *Avp* ^+^ (VEH: 4.86 ± 0.25 *vs* PNA: 5.34 ± 0.24, p = 0.21 unpaired t-test) or in *Avp* ^-^ cells (VEH *Avp* ^-^: 6.52 ± 0.74; *vs* PNA *Avp* ^-^: 6.78 ± 0.61; p = 0.79, unpaired t-test; **Figure 1D** **).**

Together, these data confirm that *Ar* is expressed in the SCN of female mice, including in a subset of *Avp*-expressing cells. Importantly, our observations also reveal that PNA increases *Ar*-expression in the adult SCN.

### 3.2 PNA treatment decreases SCN^AVP^ neuron projections to RP3V^KISS1^ neurons

**Figure 2:**
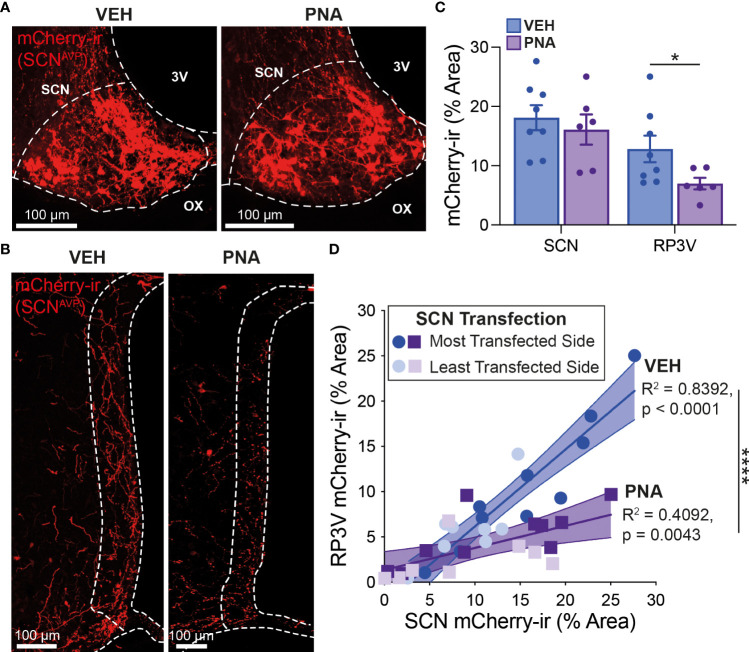
PNA decreases innervation of the RP3V by SCN^AVP^ neuron projection fibers. **(A)** Example maximum projection images showing mCherry-ir neurons (red) in the SCN of VEH and PNA Avp-cre mice injected with AAV-DIO-mCherry. White dashed lines delineate the SCN, the third ventricle (3V) and optic chiasm (OX). **(B)** Maximum projection images showing mCherry-ir SCN^AVP^ neuron fibers in the RP3V of the VEH and PNA mice shown in **(A)**. White dashed lines delineate the RP3V, 3V and OX. **(C)** Bar graphs summarizing SCN transfection (*left*) and RP3V innervation by mCherry-expressing SCN^AVP^ neuron projection fibers (*right*) in VEH and PNA Avp-cre mice with successful SCN transfection. **(D)** Linear regression analyses of mCherry-ir fiber density in the RP3V in relation to SCN transfection in VEH and PNA mice. *p < 0.05 unpaired t-test; ****p < 0.0001 ANCOVA.

We next sought to determine the impact of PNA treatment on the SCN^AVP^→RP3V^KISS1^ neuron circuit. Nine VEH and nine PNA Avp-cre females received stereotaxic injections of AAV-DIO-mCherry in the SCN. This resulted in cre-mediated recombination and expression of mCherry in AVP neurons as reported previously (53). Mice were perfused with fixative on diestrus at least 2 weeks later. Of these, eight VEH and six PNA mice had sufficient transfection in the SCN (see methods). Levels of transfection on the most transfected side of the brain, as assessed by SCN mCherry-ir, were similar in VEH (18.10 ± 2.12% of SCN area covered with mCherry-ir) and in PNA mice (16.10 ± 2.56%; p = 0.55, unpaired t-test *vs* VEH; **Figures 2A, C**). As reported previously (53), mCherry-ir projection fibers were detected in the RP3V of VEH and PNA mice (**Figure 2B**). Fiber density on the side ipsilateral to the most transfected SCN, however, was significantly lower in PNA (6.98 ± 0.98% of RP3V area covered with mCherry-ir) than in VEH mice (12.82 ± 2.25%; p = 0.04, unpaired t-test *vs* PNA; **Figure 2C**). A significant correlation was found between SCN transfection and RP3V mCherry-ir fiber innervation in VEH and PNA mice (p < 0.0001, R^2^ = 0.84 and p = 0.004, R^2^ = 0.41, respectively; linear regressions), indicating that these fibers originate in the SCN, as seen previously (52, 53). The slope of the linear regression was significantly lower in PNA than in VEH mice (0.24 [95% confidence interval 0.09-0.40] and 0.85 [95% confidence interval 0.65-1.05], respectively; n = 18 hemibrains in PNA and in VEH mice; p < 0.0001 ANCOVA; **Figure 2D**) reflecting the lower fiber density in the RP3V of PNA mice.

**Figure 3:**
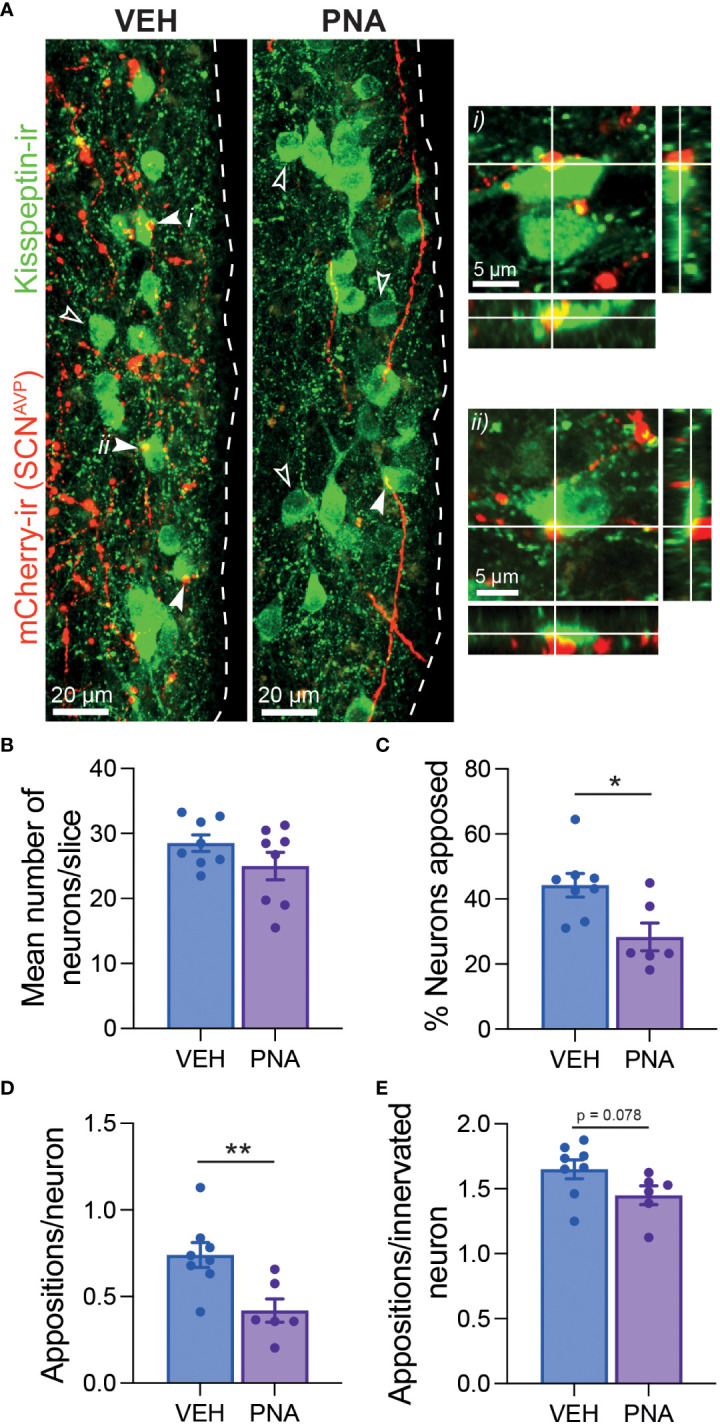
PNA reduces SCN^AVP^ neuron fiber innervation of RP3V^KISS1^ neurons. **(A)** Example maximum projection images showing mCherry-ir SCN^AVP^ neuron fibers (red) around kisspeptin-ir neurons (green) in the RP3V of VEH and PNA Avp-cre mice injected in the SCN with AAV-DIO-mCherry. Example appositions between mCherry-ir SCN^AVP^ neuron fibers and kisspeptin-ir somata are indicated with filled arrowheads. i) and ii) show single confocal planes and orthogonal views for two kisspeptin-ir neurons with appositions. Empty arrowheads point to kisspeptin-ir somata that do not receive such appositions. **(B)** Bar graph summarizing the average number of kisspeptin-ir neurons per section in the RP3V of VEH and PNA mice. **(C)** Mean proportion of kisspeptin-ir neuron somata with at least one close apposition by an mCherry-ir fiber. **(D, E)** Average numbers of appositions per kisspeptin-ir neuron **(D)** and per innervated kisspeptin-ir neuron **(E)**. *p < 0.05 and **p < 0.01 unpaired t-tests.

We then examined close appositions between mCherry-ir fibers and kisspeptin-ir RP3V somata. RP3V^KISS1^ cell counts were similar in VEH (28.52 ± 1.29 cells per slice; n = 8) and PNA mice (25.00 ± 2.12; n = 8; p = 0.18 unpaired t-test; **Figures 3A, B**). mCherry-ir fibers were seen surrounding kisspeptin-ir neurons in the RP3V of VEH and PNA mice (**Figure 3A**). The proportion of kisspeptin-ir neurons with at least one mCherry-ir fiber apposition, however, was significantly smaller in PNA (28.34 ± 4.28%, n = 6) than in VEH mice (44.26 ± 3.61%, n = 8; p = 0.015, unpaired t-test; **Figures 3A, C**). Consequently, the mean number of mCherry-ir appositions per kisspeptin-ir neuron was significantly lower in PNA (0.42 ± 0.07) than in VEH mice (0.74 ± 0.07; p = 0.008, unpaired t-test; **Figure 3D**). The number of appositions per innervated kisspeptin-ir neuron, on the other hand, tended to be lower but was not statistically different (VEH: 1.65 ± 0.07; PNA: 1.45 ± 0.07; p = 0.078, unpaired t-test; **Figure 3E**). Therefore, while the proportion of innervated kisspeptin-ir neurons is lower in PNA mice, those innervated kisspeptin neurons receive, on average, a similar number of putative inputs as in VEH mice.

Together, these observations indicate that SCN^AVP^ neuron projections to RP3V^KISS1^ neurons are reduced in PNA mice.

### 3.3 AVP excites RP3V^KISS1^ neurons to a similar extent in VEH and PNA mice

We previously reported that exogenous AVP and AVP release from SCN^AVP^ neuron projection fibers stimulate RP3V^KISS1^ neuron action potential firing (53, 65). We, thus, next examined the function of the SCN^AVP^→RP3V^KISS1^ circuit in PNA and VEH mice. These experiments were carried out in diestrous mice as proestrus was not detected in PNA mice.

**Figure 4:**
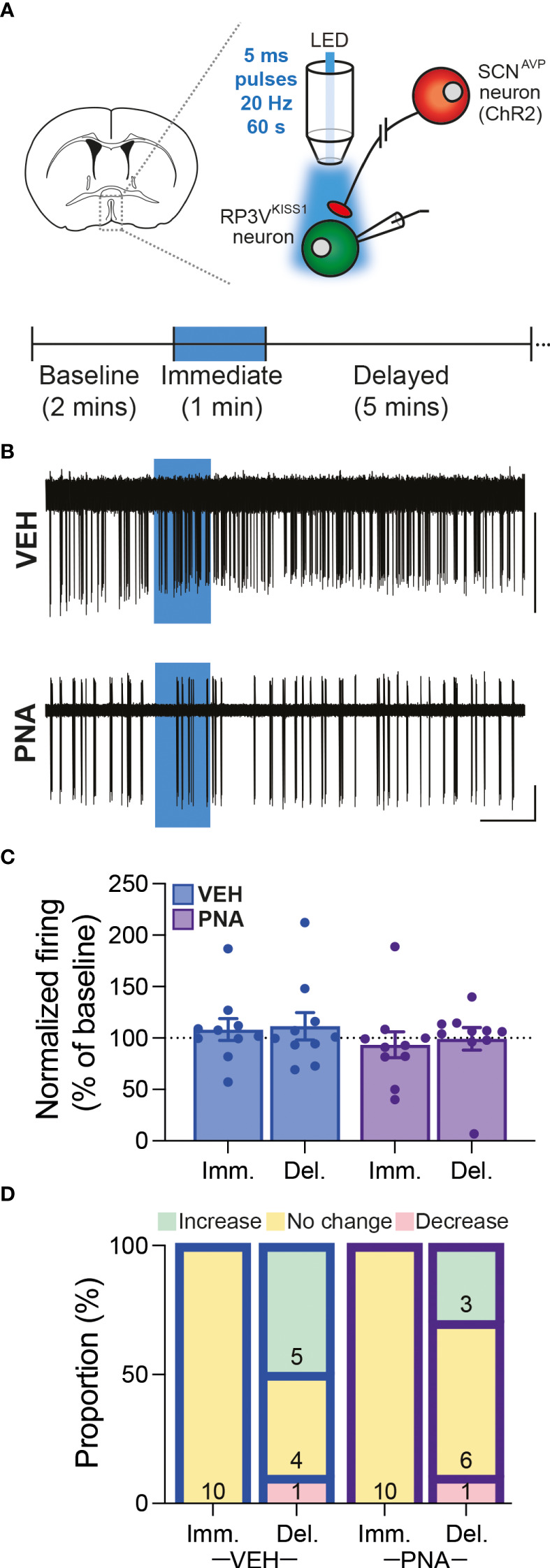
SCN^AVP^→RP3V^KISS1^ neuron circuit function in diestrous VEH and PNA mice. **(A)** Experimental set-up. Cre-dependent AAV-DIO-ChR2-mCherry vectors were injected in the SCN of Avp-cre:Kiss1-hrGFP female mice. GFP-expressing neurons were targeted for cell-attached recordings in brain slices containing the RP3V and SCN^AVP^ neuron projection fibers stimulated with trains of blue LED light pulses. The recording protocol, with timing of light stimulation and periods considered for analysis [immediate (Imm.) and delayed (Del.)], is illustrated in the lower panel. **(B)** Example traces showing spontaneous action potential firing of RP3V^KISS1^ neurons from VEH and PNA mice. Blue boxes indicate when the light stimulation occurred. **(C)** Summary graph of normalized RP3V^KISS1^ neuron firing rate during (Imm.) and within 5 minutes after (Del.) optogenetic stimulation in VEH and PNA mice. **(D)** Proportions of RP3V^KISS1^ neurons displaying responses during and after optogenetic stimulation in VEH and PNA mice. Numbers in bars are cell numbers. Scale bars = 50 pA/60 s.

**Figure 5:**
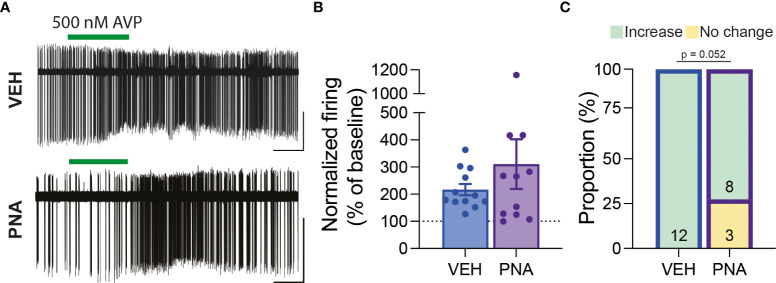
RP3V^KISS1^ neuron responses to exogenous AVP are maintained in PNA mice. **(A)** Example traces illustrating the effect of exogenous AVP (green bars) on RP3V^KISS1^ neuron action potential firing in slices from VEH (*top*) and PNA (*bottom*) mice. **(B)** Bar graph summarizing AVP-induced changes in RP3V^KISS1^ neuron firing rate in VEH and PNA mice. **(C)** Proportions of RP3V^KISS1^ neurons responding to exogenous AVP in VEH and PNA mice. Numbers in bars are cell numbers. Scale bars = 50 pA/60 s.

Overall, we observed that RP3V^KISS1^ neuron baseline spontaneous action potential firing was similar in brain slices from VEH (1.55 ± 0.28 Hz; n = 22 in 5 mice) and PNA (1.29 ± 0.30 Hz; n = 21 in 4 mice; p = 0.53, unpaired t-test) Avp-cre:Kiss1-hrGFP and Kiss1-hrGFP mice. In slices from Avp-cre:Kiss1-hrGFP mice that received SCN injections of AAV-DIO-ChR2-mCherry, ChR2-expressing SCN^AVP^ neuron projection fibers were stimulated with a train of blue light pulses (20 Hz for 60 seconds; **Figure 4A**). As reported previously (53), this had no effect on action potential firing during the stimulus, but increased firing thereafter in subsets of cells (**Figures 4B–D**). Overall, however, and in agreement with our previous observations in diestrous mice (53), optogenetic stimulation of SCN^AVP^ neuron fibers had no significant effect on RP3V^KISS1^ neuron firing in VEH or in PNA mice (**Table 1**) and neither the proportion of cells excited (VEH: 50%, PNA: 30%; p = 0.36 chi-squared test) nor the overall changes in firing (percentage of baseline firing; immediate response during stimulation, VEH: 108.10 ± 10.55% and PNA: 93.37 ± 12.66%; delayed response after stimulation, VEH: 111.3 ± 13.20% and PNA: 99.22 ± 10.99%; p = 0.38 and 0.49, respectively, unpaired t-tests; n = 10 in 3 mice each; **Figures 4B–D**) significantly differed in VEH and PNA mice. Similarly, exogenous AVP (500 nM) stimulated RP3V^KISS1^ neuron action potential firing (**Figure 5A**) as seen previously (53, 65), and these responses were comparable in VEH and PNA mice (normalized to baseline firing; VEH: 216.70 ± 20.80% and PNA: 310.80 ± 91.48%; p = 0.31 unpaired t-test; n = 12 in 3 VEH mice and 11 in 3 PNA mice; **Figure 5B**; **Table 2**). The proportion of RP3V^KISS1^ neurons excited by AVP appeared smaller in PNA mice, but this did not reach significance (72.70% in PNA and 100.00% in VEH; p = 0.052, chi-squared test; **Figure 5C**).

**Table 1:** Summary of RP3V^Kiss1^ neuron responses to optogenetic stimulation of SCN^AVP^ neuron projection fibers in VEH and PNA mice.

	VEH	PNA
Baseline firing (Hz)	2.10 ± 0.48	0.97 ± 0.41
Immediate effect (Hz)	2.23 ± 0.49	0.94 ± 0.41
Delayed effect (Hz)	2.12 ± 0.42	0.95 ± 0.41
One-way ANOVA p value	0.63	0.85
n (cells)	10	10
N (mice)	3	3

**Table 2:** Summary of RP3V^Kiss1^ neuron responses to exogenous AVP in VEH and PNA mice.

	VEH	PNA
Baseline firing (Hz)	1.11 ± 0.28	1.60 ± 0.43
Delayed effect (Hz)	2.50 ± 0.76	3.15 ± 0.81
Paired t-test p value	0.029*	0.020*
n (cells)	12	11
N (mice)	3	3

*statistically significant

Together, these findings indicate that PNA did not measurably change the function of the SCN^AVP^→RP3V^KISS1^ neuron circuit at baseline, in diestrous mice.

## 4 Discussion

We explored the possibility that the circuits that control ovulation in females are altered in a PNA mouse model of PCOS. Here, we report that a proportion of SCN cells express *Ar* mRNA. This includes SCN *Avp*-expressing cells, some of which may be involved in the circuit that controls the preovulatory LH surge through their projections to RP3V^KISS1^ neurons (51–53). Importantly, we observed that prenatal androgen treatment results in increased *Ar* expression in the SCN of adult female mice. This indicates that SCN cells, including SCN^AVP^ neurons, may be responsive to circulating androgens and, moreover, that PNA might increase the sensitivity of subsets of SCN cells to circulating androgens. This is associated with evidence for fewer fiber projections from SCN^AVP^ neurons to the RP3V and for fewer RP3V^KISS1^ neurons receiving innervation from SCN^AVP^ neurons in PNA mice. In addition to anatomical alterations of the SCN^AVP^→RP3V^KISS1^ neuron circuit, the absence of estrous cycles likely prevented the expression of the estrous cycle-dependent plasticity that drives kisspeptin neuron activity on proestrus (53). The functionality of the circuit at baseline, however, was not measurably affected. Together, these observations reveal that PNA promotes alterations in gene expression and efferent projections within a circuit involved in regulating the preovulatory surge. These alterations might contribute to ovulatory dysfunction in this model of PCOS.

### 4.1 SCN *Ar* expression

We observed expression of the *Ar* gene in the SCN of VEH and PNA female mice. This is consistent with previous studies that reported *Ar* mRNA and AR-ir in the SCN of female rats and mice (56, 57, 66). In female rats, AR-ir is found primarily in the core SCN, with little immunoreactivity in AVP neurons (56). We find here, however, that greater than 35% of *Avp*-expressing SCN cells also express *Ar* in female mice. This could reflect expression of *Ar* at levels too low for IHC detection of AR-ir. This might also be due to species differences as it was previously reported that the mouse SCN displays greater numbers of AR-ir cells than the rat SCN (57). The identity of *Avp*-negative SCN cells that express *Ar* is unknown. Among the possible subsets of SCN cells, it is tempting to speculate that some of these are vasoactive intestinal peptide-expressing neurons, which project directly to GnRH neurons (47), or prokineticin 2-expressing neurons, which might play a role in the LH surge (67). Further experiments will be required to fully characterize SCN *Ar*-expressing neurons.

Along with these previous reports, our finding that *Ar* is expressed in the SCN of female mice, including in SCN^AVP^ neurons, suggests that the SCN may be sensitive to variations in androgen levels. Elevated circulating testosterone seen in PNA mice (14, 59) might, therefore, have an impact on SCN cells. Consistent with this idea, we observed increased *Ar* expression in the SCN of PNA mice. A similar trend was seen in the number of *Avp*-positive SCN cells expressing *Ar*, although this did not reach statistical significance. Interestingly, PNA also increases AR expression in the RP3V (16), a region of the hypothalamus involved in the preovulatory surge (68), and we note that RP3V^KISS1^ neurons do express *Ar* (54). Whether AR expression is altered specifically in RP3V^KISS1^ neurons in PNA mice, however, remains to be seen. Together with the results of our RNAscope^®^ experiment, these observations suggest that PNA might increase sensitivity to circulating androgens within the circuits that initiate the preovulatory surge.

The role of AR in SCN function in females is unclear. In male rats, androgen receptor activation restores gonadectomy-induced alterations in circadian activity rhythms (56). In PNA females, however, excess androgen is not associated with impaired circadian wheel running or with alteration in SCN clock gene expression rhythms. Interestingly, however, PNA impairs the coordination between SCN rhythms and peripheral clock rhythms, suggesting that the synchronizing output of the SCN might be altered (69). Curiously, the link between circadian function and excess androgen might be bidirectional as chronic exposure to constant light results in PCOS-like symptoms, including elevated testosterone secretion, in rats (70).

### 4.2 SCN^AVP^→RP3V^KISS1^ neuron circuit in adult PNA females

Using AAV-mediated anterograde tract-tracing, we observed here that the projections of SCN^AVP^ neurons to RP3V^KISS1^ neurons, observed previously by us and others (51–53), are substantially reduced in adult PNA female mice. Decreased innervation of RP3V^KISS1^ neurons by SCN^AVP^ neurons is unlikely to result from PNA-induced alterations in SCN AVP expression. First, *Avp* gene expression was not measurably affected by PNA in our RNAscope^®^ experiment, where we counted similar numbers of *Avp*-expressing neurons with comparable numbers of mRNA particles in VEH and PNA. Second, in our anterograde tract-tracing experiment, transfection of cre-expressing SCN^AVP^ neurons with AAV-DIO-mCherry was comparable between VEH and PNA. The decreased density of mCherry-ir fibers in the RP3V and numbers of close appositions to RP3V^KISS1^ neurons therefore likely reflect decreased axonal projections from SCN^AVP^ neurons.

PNA and other preclinical PCOS animal models have revealed alterations in afferent circuits to GnRH neurons and to kisspeptin neurons in the arcuate nucleus and POA of rodents and sheep (12, 14–17, 71, 72). Our finding that SCN^AVP^ neuron projections to RP3V^KISS1^ neurons are reduced in PNA mice is reminiscent of observations made in sheep in which innervation of POA kisspeptin neurons is decreased in adult PNA ewes (17), although brain areas where this innervation originates are likely distinct in sheep and in rodents, perhaps reflecting the independence of the LH surge from time-of-day signals in the sheep (73). In addition, previous studies of mouse arcuate nucleus kisspeptin neurons revealed that PNA is associated with decreased glutamatergic and γ-aminobutyric acid (GABA)ergic innervation as well as reduced input from several hypothalamic areas (15), suggesting that both hypothalamic kisspeptin neuron populations might be dysregulated. On the other hand, PNA increases GABAergic synaptic input to GnRH neurons (14, 16). The functional significance of this differential pattern of rewiring to GnRH and kisspeptin neurons by PNA is unknown.

Despite clear anatomical alterations in the projections of SCN^AVP^ neurons to RP3V^KISS1^ neurons, we did not detect any measurable changes in the function of this circuit. First, spontaneous action potential firing of RP3V^KISS1^ neurons was similar in brain slices from VEH and PNA mice. This is consistent with a recent report that PNA does not have a measurable impact on arcuate nucleus kisspeptin neuron firing (74) but in sharp contrast with previous observations that GnRH neuron firing is substantially increased by PNA (13, 75). These findings suggest that PNA may not directly alter baseline excitability in kisspeptin neurons as it does in GnRH neurons. Second, we observed that RP3V^KISS1^ neuron action potential firing was stimulated by AVP to a similar extent in VEH and PNA mice. This indicates that AVP to vasopressin 1a receptor signaling in RP3V^KISS1^ neurons (53, 65) is not substantially altered in PNA animals. Further, using selective optogenetic stimulation of SCN^AVP^ neuron projections to RP3V^KISS1^ neurons, we observed that PNA did not change the function of this circuit during diestrus. We have previously reported that the output of the SCN^AVP^→RP3V^KISS1^ neuron circuit varies considerably across the estrous cycle, driving kisspeptin neuron activity on proestrus but not on diestrus (53). It is, therefore, possible that PNA, in addition to altering SCN *Ar* expression and impairing the projections from SCN^AVP^ neurons to RP3V^KISS1^ cells, prevents the full expression of plasticity within this circuit by disrupting estrous cycles. We note that changes in this circuit might also affect female sexual behavior, as RP3V^KISS1^ neurons also regulate lordosis in rodents (76).

### 4.3 Ontogeny of PNA-induced impairments in the SCN^AVP^→RP3V^KISS1^ neuron circuit

Although early neonatal administration of androgen or estrogen hormones to female rats potently suppresses *Kiss1* expression in the preoptic area (77, 78), the impact of exposure to elevated androgens on RP3V^KISS1^ neuron numbers and/or *Kiss1* expression is variable in animal models of PCOS [reviewed in (79)]. In the RP3V of adult PNA female rodents, *Kiss1* expression is unaltered (18, 55) while numbers of kisspeptin-ir neurons are reported as similar or decreased (18, 19). We find here, using immunofluorescence, that numbers of RP3V^KISS1^ neurons are similar in adult diestrous VEH and PNA females. This suggests that PNA does not affect the development and/or maintenance of kisspeptin expression in the RP3V, despite their expression of *Ar* (54). It is worth noting, however, that *Kiss1* mRNA and kisspeptin are first expressed in the RP3V postnatally (26, 80, 81), days after the initial exposure to androgens *in utero*, and that RP3V^KISS1^ neuron numbers reach adult-like levels (26) prior to or around the time of the observed increase in circulating testosterone in postnatal PNA females (71), providing a potential explanation for our observations.

Exposure to androgens *in utero* results in changes in GnRH neuron gene expression, in reprogramming of estrogen and progesterone negative feedback and in rewiring of GABAergic inputs to GnRH neurons prior to puberty (16, 59, 71, 82). In contrast, other pieces of evidence support the presence of post-pubertal effects of elevated testosterone in adult female PNA mice. Administration of an AR antagonist to adult PNA female mice indeed restores normal GABAergic synaptic input to GnRH neurons and estrous cyclicity (14, 71), whereas ovariectomy reverses and DHT restores elevated GnRH neuron action potential firing in adult PNA mice (75). The timeline of development of SCN^AVP^ neuron projections to RP3V^KISS1^ neurons is currently unknown. We speculate, however, that establishment of these projections might occur concurrently with the pubertal increase in kisspeptin expression in the RP3V, in parallel with the increase in VIP innervation of GnRH neurons seen in female rats (83) and in preparation for the first estrus. If this were the case, it would suggest that the effects of PNA and elevated androgens on this circuit might be occurring after the pubertal transition as circulating testosterone becomes significantly elevated sometime between postnatal day 40 and 50 (71). Supporting this idea, ovariectomy and estrogen replacement in ovariectomized PNA females restores positive feedback and the LH surge (59), suggesting that abnormal sex steroid secretion, possibly including testosterone, by the adult PNA ovary contributes to reversibly disrupt, but not to re-program, the circuits that mediate estrogen positive feedback. Our observations suggest that this might include the SCN^AVP^→RP3V^KISS1^ neuron circuit. Lastly, despite our observations that *Ar* is expressed in the SCN, including in *Avp*-expressing cells, and that SCN *Ar* expression is increased in adult PNA females, whether or not impairments in the SCN^AVP^→RP3V^KISS1^ neuron circuit are dependent on AR activation remains to be seen. As PNA may lower circulating estrogen, although not always (16, 18, 19, 59), and estrogen replacement increases the proportion of RP3V^KISS1^ neurons innervated by AVP-ir fibers in ovariectomized mice (51), we cannot fully rule out that alterations in the SCN^AVP^→RP3V^KISS1^ neuron circuit result from PNA-induced dysregulation of estrogen positive feedback effects on this circuit. Further experiments are required to disentangle these potential effects of gonadal steroids in PNA mice.

### 4.4 Relevance to clinical PCOS

Preclinical animal models of PCOS have provided valuable insight into the pathogenesis of this disorder and progressed our understanding of its neuroendocrine underpinnings [reviewed in (8)]. While impairments in feedback regulation of pulsatile GnRH and LH secretion reported in some of these models correlate well with the pathophysiology of human PCOS, it may be argued that the impact of PNA on the circuits involved in generating the preovulatory surge in female rodents does not. Indeed, although the human preovulatory LH surge requires GnRH receptor signaling (84), GnRH might only play a permissive role in the human preovulatory LH surge (85) and GnRH secretion might, in fact, decrease during the surge (86). Moreover, it is unknown if kisspeptin neurons in the human POA (87) play any role in the preovulatory surge. On the other hand, there is evidence of positive feedback-like effects of estrogen on these neurons (35), while kisspeptin administration induces surge-like increases in LH secretion in women (88). A potential role of the central circadian clock in impaired fertility is supported by reports that circadian disruptions may negatively impact gonadotropin secretion and the menstrual cycle (89–92) and may be associated with PCOS (93). In addition, the preovulatory surge initiates preferentially in the morning in women (94–98), and is time-locked to the diurnal cortisol peak (98), which is controlled by the circadian clock (99). Further, AR expression has been reported in the SCN of men and women (100). Although it is unknown if the timing of the preovulatory surge in women involves projections from the SCN to the GnRH neuronal network and POA kisspeptin neurons, it is tempting to speculate that elevated testosterone might have an impact on the output of the central circadian clock in PCOS patients similar to what we observed in adult PNA female mice. In support of this idea, PCOS may be associated with altered melatonin secretory rhythms, which are controlled by the central circadian clock (101), and this dysregulation correlates with testosterone levels in patients (102, 103).

## 5 Conclusion

The results reported here indicate that PNA results in alterations of projections from SCN^AVP^ neurons to RP3V^KISS1^ neurons in adulthood. As this circuit is thought to play a key role in generating the preovulatory surge in female rodents [reviewed in (104)] by driving the activity of RP3V^KISS1^ neurons and, downstream, GnRH neurons (32, 53, 65), these impairments might contribute to ovulatory dysfunction in this animal model of PCOS.

## Data availability statement

The raw data supporting the conclusions of this article will be made available by the authors, without undue reservation.

## Ethics statement

The animal study was reviewed and approved by the Animal Ethics Committee, University of Otago and the Institutional Animal Care and Use Committee, Kent State University.

## Author contributions

BJ, AM, RC and RP designed the research. BJ, AM, DL and ST carried out the research. BJ, AM, RC and RP analyzed the data. AM, LC and ML contributed laboratory space, reagents and analysis tools. BJ, AM, ML, RC and RP wrote drafts of the article and approved final version to be submitted.

## Funding

This work was supported by grants from the Health Research Council of New Zealand (grant #16-027 to RP and RC) and from NICHD (grant #R00HD096120 to AM). BJ was supported by a University of Otago PhD Scholarship.

## Acknowledgments

We thank members of the Piet and Campbell laboratories for their comments on earlier drafts of this manuscript. Publication of this study was supported by the Department of Biological Sciences and the Open Access Publishing Fund at Kent State University.

## Conflict of interest

The authors declare that the research was conducted in the absence of any commercial or financial relationships that could be construed as a potential conflict of interest.

## Publisher’s note

All claims expressed in this article are solely those of the authors and do not necessarily represent those of their affiliated organizations, or those of the publisher, the editors and the reviewers. Any product that may be evaluated in this article, or claim that may be made by its manufacturer, is not guaranteed or endorsed by the publisher.
